# Clinical Outcome after Intra-Arterial Stroke Therapy in the Very Elderly: Why is it so Heterogeneous?

**DOI:** 10.3389/fneur.2014.00060

**Published:** 2014-04-29

**Authors:** Ronil V. Chandra, Thabele M. Leslie-Mazwi, Brijesh P. Mehta, Albert J. Yoo, Claus Z. Simonsen

**Affiliations:** ^1^Diagnostic and Interventional Neuroradiology, Monash Health, Monash University, Melbourne, VIC, Australia; ^2^Neuroendovascular and Neurologic Critical Care, Massachusetts General Hospital, Harvard Medical School, Boston, MA, USA; ^3^Neuroendovascular and Neuroradiology, Massachusetts General Hospital, Harvard Medical School, Boston, MA, USA; ^4^Department of Neurology, Aarhus University Hospital, Aarhus, Denmark

**Keywords:** endovascular procedures, stroke, thrombolysis, elderly, intra-arterial therapy

## Abstract

Very elderly patients (i.e., ≥80 years) are disproportionally affected by acute ischemic stroke. They account for a third of hospital stroke admissions, but two-thirds of overall stroke-related morbidity and mortality. There is some evidence of clinical benefit in treating selected very elderly patients with intravenous thrombolysis (IVT). For very elderly patients ineligible or non-responsive to IVT, intra-arterial therapy (IAT) may have promise in improving clinical outcome. However, its unequivocal efficacy in the general population remains to be proven in randomized trials. Small cohort studies reveal that the rate of good clinical outcome for very elderly patients after IAT is highly variable, ranging from 0 to 28%. In addition, they experience higher rates of futile reperfusion than younger patients. Thus, it is imperative to understand the factors that impact on clinical outcome in very elderly patients after IAT. The aim of this review is to examine the factors that may be responsible for the heterogeneous clinical response of the very elderly to IAT. This will allow the reader to integrate the current available evidence to individualize intra-arterial stroke therapy in very elderly patients. Placing emphasis on pre-stroke independent living, smaller infarct core size, short procedure times, and avoiding general anesthesia where feasible, will help improve rates of good clinical outcome.

## Introduction

Almost one-third of new ischemic strokes occur in patients aged 80 years or older (1). In this very elderly cohort, ischemic stroke is associated with more severe neurological impairment and higher morbidity and mortality rates, compared to non-elderly patients (2). The Third International Stroke Trial (IST-3) demonstrated the likely clinical benefit of intravenous thrombolysis (IVT) in very elderly stroke patients (3). For patients ineligible or non-responsive to intravenous thrombolysis, intra-arterial therapy (IAT) may have promise in improving clinical outcome. However, its efficacy in younger stroke patients remains to be proven in randomized trials. Cohort studies reveal that the rate of good clinical outcome after very elderly patients are treated with IAT is heterogeneous, ranging from 0 to 28% (4–10). In addition, close to 50% of elderly patients experience futile reperfusion, i.e., 90-day follow-up modified Rankin scale (mRS) score of 3–6, despite thrombolysis in cerebral infarction (TICI) 2B-3 reperfusion and no intracranial hemorrhage (11). This is almost double the rate of futile reperfusion for younger patients. These facts could lead to treating physician reluctance to offer very elderly patients intra-arterial stroke therapy. Thus, to individualize intra-arterial stroke therapy decisions in very elderly patients, there is a critical need to understand the factors that impact on clinical outcome.

The aim of this focused review is to examine the factors that may be responsible for this heterogeneity in clinical outcome in this growing population of very elderly stroke patients undergoing IAT. This will allow the reader to integrate the current available evidence with their own clinical expertise and patient wishes to make the most appropriate clinical decision for very elderly patients being considered for intra-arterial stroke therapy.

## Why Examine Outcomes in Very Elderly Patients?

The worldwide incidence of first-ever stroke in 2005 was estimated at 16 million, with 5.7 million stroke-related deaths (12). This is expected to rise to 23 million strokes and 7.8 million deaths by 2030 (12). This increase is largely driven by the rapidly growing elderly population – in the United States, the population aged greater than 85 years is expected to increase fivefold by 2050 (13). These expanding numbers of the very elderly will produce an increasing burden of age-associated disease, mandating a clear understanding of treatment options for this population.

After ischemic stroke admission, very elderly patients experience greater disability and are less likely to be discharged home, even if they receive similar rates of stroke care quality metrics as younger patients (14). Despite treatment with either IVT or IAT, there remain higher rates of in-hospital mortality than younger patients (15). Even after adjustment for differences in stroke risk factors and comorbidities, they continue to have poorer functional outcomes (2). Ultimately, elderly patients account for only one-third of all admissions but almost two-thirds of all stroke deaths (2). This disproportionate effect of ischemic stroke on the elderly will continue to grow significantly in the future.

## What have We Learned Lately about Acute Stroke Therapy in Elderly Patients?

For many years, there has been a paucity of high quality evidence for acute stroke therapy in elderly patients. Only 42 elderly patients were included in The National Institute of Neurological Disorders and Stroke (NINDS) intravenous tissue plasminogen activator (ivtPA) trial (16, 17) and they were excluded from the large IVT trials such the European Cooperative Acute Stroke Study (ECASS) III (18) and Safe Implementation of Thrombolysis in Stroke-Monitoring Study (SITS-MOST) (19).

A previous non-randomized controlled comparison of the SITS-International Stroke Thrombolysis Registry and Virtual International Stroke Trials Archive included 3,439 very elderly (age >80) patients (total *n* = 29228) (20). Although increasing age was associated with poorer outcome, the association between thrombolysis and improved outcome was maintained in very elderly patients. Recently, the results of the IST-3, an international multicenter randomized controlled trial of IVT within 6 h of ischemic stroke onset, were published (3). This included the largest cohort of elderly patients in a randomized controlled IVT trial to date (age >80; *n* = 1617; total *n* = 3035). Although IST-3 failed to reach its primary endpoint, secondary analysis revealed that treatment benefit was greatest in the 0–3 h cohort, with 30.6% of those treated with ivtPA alive and independent at 6 months compared to 22.7% of controls. The vast majority (*n* = 726/849) included in this 0–3 h cohort were >80 years, and the treatment benefit in patients >80 years was at least as large as younger patients. Thus, potential treatment benefit from IVT does likely extend to elderly patients.

In contrast, there is no high quality randomized controlled data reporting on clinical outcomes of IAT in the elderly. The large IAT trials, including the recent three trials published in the New England Journal of Medicine, all excluded elderly patients greater than 85 years or did not reported age-related outcomes (21–28). The smaller case cohort studies have all reported quite variable clinical outcomes. As defined by 90-day mRS ≤2, the rates of good clinical outcome in elderly patients range from 0 to 28% (4–10). Table 1 summarizes data from current published studies designed to assess clinical outcome after very elderly patients with anterior circulation stroke are treated with IAT. These studies include patients treated with intra-arterial thrombolysis, and older generation mechanical devices such as the Merci Retrieval System (Concentric Medical, Mountain View, CA, USA) and the Penumbra System (Penumbra, Alameda, CA, USA) that have lower reperfusion rates compared to modern intra-arterial stentrievers.

**Table 1:** **Summary of current published studies designed to assess clinical outcome after very elderly patients with anterior circulation stroke undergo IAT**.

Author (year)	Age used to define elderly cohort	Number of very elderly patients	Number of younger controls	sICH rate in elderly cohort	Primary outcome measures	Summary of primary outcome
Singer et al. (11)	77	Registry of 362 patients; 25% of cohort >76; 91 patients	Registry of 362 patients; 75% of cohort 18–76 years; 271 patients	Not reported; 3% ECASS parenchymal hematoma type II in total cohort	90-day mRS and “futile recanalization”	Outcome is age-dependent with elderly patients (in the highest age quartile of 77–94 years) having the lowest rates of good outcome (mRS 0–2) of 17% compared to 60% for patients in the lowest age quartile (18–56 years). Elderly (77–94 years) patients had higher rates (46 vs. 24%) of clinically “futile recanalization” (defined as 90-day mRS score ≥3 despite successful recanalization and no subsequent hemorrhage) compared the lowest age quartile (18–56 years)
Kurre et al. (7)	80	109	0	6% ECASS parenchymal hematoma type II	90-day mRS	13% had pre-stroke disability of mRS score 3–4. By 90 days, 17% of elderly patients had a mRS score 0–2; mortality rate was 48%
Willey et al. (15)	80	186	622	Not reported	In-hospital mortality	Very elderly patients had a higher risk of in-hospital mortality compared with younger counterparts regardless of treatment modality (IAT and/or IVT) (OR, 2.13; 95% CI, 1.60–2.84). IAT does not increase the risk of in-hospital mortality in very elderly patients compared to IVT alone
Chandra et al. (5)	80	49	130	4% ECASS parenchymal hematoma type II	90-day mRS and mortality	Very elderly patients had significantly lower rates of good outcome (mRS 0–2: 2 vs. 33%; *P* < 0.0001) and higher mortality (59 vs. 24%; *P* < 0.0001) at 90 days compared to younger patients
Mono et al. (9)	80	43	524	2%	90-day mRS and mortality	Very elderly patients had significantly lower rates of good outcome (mRS 0–2: 28 vs. 46%; *P* = 0.019) and higher mortality (40 vs. 22%; *P* = 0.0008) at 90 days compared to younger patients
Ghobrial et al. (29)	75	51 patients aged 75 or greater	0	6%	Discharge mRS	At time of discharge, 33% of elderly patients had a mRS score 0–3; mortality rate at discharge was 22%
Arkadir et al. (4)	80	14	66	7%	90-day mRS	Very elderly patients had significantly lower rates of good outcome (mRS 0–2: 0 vs. 41%; *P* = 0.008)
Mazighi et al. (8)	80	25	59	12%	90-day mRS	Very elderly patients had significantly lower rates of good outcome (mRS 0–2: 28 vs. 64%; *P* = 0.002) and higher mortality (40 vs. 22%; *P* = 0.002) at 90 days compared to younger patients
Loh et al. (30)	80	31	75	11%	mRS at discharge and stroke-related death	Very elderly patients had lower rates (not statistically significant) of good outcome (mRS 0–2: 19 vs. 33%; *P* = 0.17) at discharge compared to younger patients. Rates of stroke-related death were significantly higher in elderly patients (48 vs. 15%; *P* = 0.0005) at discharge compared to younger patients
Qureshi et al. (10)	80	24	77	8%	30–90 day mRS and mortality	Very elderly patients had lower rates (not statistically significant) of good outcome (mRS 0–2: 21 vs. 38%; *P* = 0.21) at 30–90 days compared to younger patients. There were higher rates of mortality (54 vs. 29%; *P* = 0.02) as compared to younger patients
Kim et al. (6)	80	33	81	7%	90-day mRS	Very elderly patients had significantly lower rates of good outcome (mRS 0–2: 26 vs. 40%; *P* = 0.02) and higher mortality (43 vs. 20%; *P* = 0.01) at 90 days compared to younger patients

*All studies included small numbers of posterior circulation stroke patients, with the exception of Singer et al. (11), Chandra et al. (5), and Arkadir et al. (4) who only included anterior circulation stroke*.

There is a paucity of data on outcomes of elderly patients treated with these stentriever devices – the Solitaire With Intention for Thrombectomy (SWIFT) and Trevo vs. Merci retrievers for thrombectomy revascularization of large vessel occlusions in acute ischemic stroke (TREVO2) trials excluded patients >85 years, and no age-stratified outcomes for older patients have been reported (31, 32). The large prospective single-arm study of patients with large vessel anterior circulation strokes treated using the solitaire FR stentriever also excluded patients aged >84 years (33). Similarly, the prospective European TREVO trial excluded those aged >85 years (34).

Notably, a recent abstract published from the North American Solitaire Stent Retriever Acute Stroke registry did include 64 very elderly patients and compared outcomes to 245 non-elderly patients (35). Despite similar rates of reperfusion (TICI ≥ 2a) and symptomatic intracranial hemorrhage (sICH), very elderly patients had significant lower rates of good clinical outcome (90-day mRS ≤2: 27 vs. 45%; *P* = 0.007) and higher mortality (42 vs. 27%: *P* = 0.03) compared to younger patients. The Trevo Retriever Registry has also been recently launched in the US to assess the real world performance of the Trevo Retriever. There is no upper age limit, and this will provide further data for the very elderly cohort.

## Why are Clinical Outcomes Heterogeneous?

There are many factors that impact on the clinical outcome of patients undergoing IAT. In elderly patients, pre-stroke disability, baseline infarct size, cerebral collateral circulation, use of general anesthesia, and procedural time are prominent factors. In addition, there are concerns that very elderly patients have higher rates of post-treatment hemorrhage, and significant differences in post-treatment care that could negatively impact on clinical outcome. So what have we learnt lately?

### Pre-stroke disability

Pre-stroke disability and institutionalization (not living at home) are independent predictors of post-stroke death and disability in elderly stroke patients (1, 36). These are common in the elderly stroke cohort – almost half of elderly patients hospitalized for index stroke have a pre-stroke mRS of 2–5, while one in seven are institutionalized prior to admission (1). In spite of these facts, most studies of IAT in elderly patients have not reported rates of baseline disability and institutionalization. Approximately, one-third of the single center elderly cohort reported by Chandra et al. had baseline mRS >1; 2% of patients had a good clinical outcome (5). Thirteen percent of the cohort examined by Kurre et al. had pre-stroke mRS scores of 3–4; 17% of the overall cohort had a good clinical outcome (7). Patients with baseline mRS scores >1 were excluded from the cohort by Mono et al.; 28% of elderly patients had a good clinical outcome (9). Nevertheless, elderly patients had significantly lower rates of good clinical outcome and higher rates of mortality than younger patients in both cohorts, suggesting that there are additional factors responsible for the worse clinical outcomes among the very elderly.

### Baseline infarct size

There are emerging data that elderly stroke patients require smaller baseline infarcts to achieve good clinical outcome when compared to younger patients. In patients undergoing IAT, final infarct volume is a powerful predictor of clinical outcome (37, 38). With increasing age, the post-treatment infarct volume that predicts good clinical outcome reduces (39). Target cut-off infarct volume for predicting a good clinical outcome calculated from 24-h post procedure CT scans suggests volumes of approximately <50 cc for patients <70 years; <30 cc for patients 70–79 years; and <15 cc for those ≥80 years (39). This is not surprising as elderly patients have lesser neurological rehabilitation potential (40) and higher rates of in-hospital medical complications after stroke, which is a predictor for death and disability in elderly patients (36). None of the current studies of IAT in the elderly report on exact baseline infarct volumes. While intra-arterial selection criteria based on baseline MR imaging may be able to accurately stratify these volume thresholds, this is unlikely to occur with sufficient accuracy using non-contrast CT or CT perfusion-based intra-arterial selection strategies, particularly early after symptom onset (41, 42).

### Collateral circulation

There are conflicting data on whether elderly stroke patients with anterior circulation occlusion have poorer leptomeningeal collaterals than younger patients. Collateral circulation can sustain the ischemic brain tissue after vessel occlusion, and determines how quickly an infarct grows to complete its course. Patients with good collaterals typically have smaller baseline infarcts and higher rates of recanalization if treated with IAT (43, 44). If successful reperfusion occurs (TICI 2b-3), infarct growth is attenuated, and a higher rate of good clinical outcome can be achieved (43, 44). It has also been reported that every 10-year increment in age almost doubles the odds of inadequate collateral circulation (45). Providing further support for this hypothesis of poorer collaterals in older patients, collateral augmentation improved outcome in elderly stroke patients in the recent safety and efficacy of NeuroFlo Technology in ischemic stroke trial (46). A good clinical outcome at 90 days was two to four times more likely in elderly patients treated with intermittent partial aortic balloon occlusion to augment cerebral blood flow and collateral circulation in a sub-group of patients older than 70 years. There was greater benefit for patients aged 80 years and older, while no benefit was observed in patients younger than 70 years. This demonstrates the importance of the collateral circulation, particularly in the elderly stroke patient.

### Anesthesia

The use of general anesthesia has been linked to poorer outcomes in patients undergoing IAT (47, 48) which may be accentuated in elderly patients. The exact mechanism of poorer outcome after general anesthesia is unclear. There is a major confounder in the data published, as baseline National Institutes of Health Stroke Scale (NIHSS) scores were higher in the general anesthesia cohort. However, in a sub-group analysis of 494 patients (total *n* = 1079), controlling for NIHSS, general anesthesia remained a predictor for poor outcome (47). The major postulate to explain this phenomenon is post-induction or intra-procedural reduction in blood pressure impacting on cerebral collateral perfusion. This is supported by the recent report that maintaining a lowest systolic blood pressure of >140 mmHg is an independent predictor of good neurological outcome after IAT (49). Post-induction hypotension occurs more often in older patients, which could contribute to poorer outcomes (50). In current reports of the elderly undergoing IAT, the rate of usage of general anesthesia range from 40 to 100% (5, 9, 11, 29), so this may be a significant factor affecting the currently published rates of good outcome.

Furthermore, it has also been shown that early neurocognitive dysfunction is common in patients undergoing carotid endarterectomy under general anesthesia (51). This phenomenon occurs at a significantly higher rate in older patients (51). Interestingly, elevation of intra-operative mean arterial blood pressure during the cross-clamp period during endarterectomy significantly reduced the incidence of this phenomenon, presumably by optimization of cerebral collateral perfusion (52). Thus, the elderly stroke patient with a large vessel occlusion may be particularly vulnerable to the negative effects of hypotension and general anesthesia during IAT.

### Procedural time

Vascular tortuosity and atherosclerotic burden in the elderly stroke population may contribute to longer procedural times that in turn negatively impacts on clinical outcome. Although no study has found a statistically significant difference in symptom onset to start or conclusion of IAT in elderly patients compared to younger patients (5, 6, 9, 30), older age is associated with longer time to access the target carotid artery in anterior circulation stroke (53). In the quarter of the total cohort (*n* = 130) where vessel tortuosity resulted in greater than 30 min delay in placing the guide catheter into the target carotid artery, there was a significant reduction in the rate of reperfusion and good neurological outcome compared to those with faster target vessel access. Notably, there was no difference in the time from symptom onset to final reperfusion.

### Post-treatment hemorrhage

There is often concern that elderly patients have higher rates of post-procedural hemorrhage compared to younger patients that could lead to futile reperfusion by worsening patient outcome or causing in-hospital mortality. However, in the cohort studies of elderly patients undergoing IAT, there were no significant differences in the rates of parenchymal hematoma (PH) type 2 [ECASS definition (54)] or sICH (5, 6, 8, 9, 30). The concern may be in part due to the variability in definitions used in these studies and the small patient numbers. In an analysis of greater than 22,000 patients undergoing IVT, although there was no difference in the rates of elderly patients experiencing sICH according to the SITS-MOST definition, the rates of sICH according to the NINDS definition were significantly higher (20). Nonetheless, in a large cohort study of anterior circulation stroke patients undergoing IAT (*n* = 1122), age was not a predictor of intracranial hemorrhage after IAT (55). Furthermore, in a large study of IAT in elderly patients (*n* = 1182), IAT did not increase the rates of in-hospital mortality compared to intravenous thrombolysis alone (15). Thus, it does not seem that the worse clinical outcomes and higher mortality rates seen in elderly patients are attributable to post-procedural hemorrhage.

### Post-treatment care

Elderly patients are more likely to have greater neurological impairment, swallowing difficulty, confusion, and urinary incontinence during hospitalization for acute stroke compared to younger patients (1). These are all risk factors for the development of pneumonia, which accounts for up to third of in-hospital deaths after ischemic stroke (56). In addition, elderly patients often have “do not resuscitate (DNR)” orders in place. In spite of these referring to patient wishes regarding cardiopulmonary resuscitation, the presence of these orders also leads to an overall reduction in physician willingness to treat (57). After hemorrhagic stroke, the presence of a DNR order leads to significantly reduced rates of guideline recommended care, including stroke unit care (58). For the elderly patient experiencing a severe stroke, the development of pneumonia or lack of guideline recommended care could contribute to poorer outcomes compared to younger patients.

## Future Directions

The reality is that an ischemic stroke occurring in an elderly patient is a severe life-threatening event. After their first-ever ischemic stroke, a third of elderly patients are likely to be dead within 30 days (59). Even after 12 months, only a quarter have improved to mild or no disability. In light of the frailty of the elderly population, we may be unrealistic in our expectations that a high proportion of elderly patients will have minimal disability after IAT. The data that are currently available suggest that the clinical outcomes in elderly patients undergoing IAT are heterogeneous, however rates of no or mild disability as high as 28% have been reported. Moreover, performing IAT in the elderly has not raised safety concerns, particularly with regard to post-treatment hemorrhage.

In light of the findings summarized in this review, individualized patient selection for IAT appears more important in the elderly stroke patient. Emphasis needs to be placed on pre-stroke independent living, smaller infarct core size, short procedure times, and avoiding general anesthesia where feasible. Multidisciplinary screening and treatment protocols for post-stroke pneumonia with minimization of ventilator time will be also important in optimizing outcomes. In the future, invasive techniques for collateral augmentation may be found to have particular benefit in the elderly. Most importantly, while we must be realistic in our expectations of clinical outcome, we should also not deny elderly patients’ guideline recommended care. As selection criteria for IAT in the elderly are refined, and with more rapid and complete reperfusion, the clinical outcome of elderly patients treated with IAT should improve.

## Conflict of Interest Statement

The authors declare that the research was conducted in the absence of any commercial or financial relationships that could be construed as a potential conflict of interest.

## Abbreviations

DNR, do not resuscitate; ECASS, European Cooperative Acute Stroke Study; IAT, intra-arterial therapy; IST-3, Third International Stroke Trial; IVT, intravenous thrombolysis; ivtPA, intravenous tissue plasminogen activator; mRS, modified Rankin scale; NIHSS, National Institutes of Health Stroke Scale; NINDS, National Institute of Neurological Disorders and Stroke; sICH, symptomatic intracranial hemorrhage; SITS-MOST, Safe Implementation of Thrombolysis in Stroke-Monitoring Study; SWIFT, Solitaire With Intention for Thrombectomy; TICI, thrombolysis in cerebral infarction; TREVO2, Trevo vs. Merci retrievers for thrombectomy revascularization of large vessel occlusions in acute ischemic stroke.
